# Retraction: Netrin-1 promotes gastric cancer cell proliferation and invasion via the receptor neogenin through PI3K/AKT signaling pathway

**DOI:** 10.18632/oncotarget.28804

**Published:** 2025-12-31

**Authors:** Kai Yin, Linjun Wang, Xuan Zhang, Zhongyuan He, Yiwen Xia, Jianghao Xu, Song Wei, Bowen Li, Zheng Li, Guangli Sun, Qing Li, Hao Xu, Zekuan Xu

**Affiliations:** ^1^Department of General Surgery, The First Affiliated Hospital of Nanjing Medical University, Nanjing, Jiangsu, China; ^2^Department of General Surgery, Affiliated Hospital of Jiangsu University, Zhenjiang, Jiangsu, China; ^3^Department of Hepatobiliary Surgery, Wuhu No.2 People ‘s Hospital, Wuhu, Anhui, China; ^4^Collaborative Innovation Center For Cancer Personalized Medicine, Nanjing Medical University, Nanjing, Jiangsu, China; ^*^These authors contributed equally to this work

**This article has been retracted:** Oncotarget has completed its investigation of this paper. Image forensic analysis confirmed that Figures 2, 3, 4, and 6 contain overlapping images. Additionally, two years after the publication of the Oncotarget paper, one of the panels in Figure 2F was duplicated in Figure 3A of reference [1], where Xu Zekuan was a corresponding author. The National Natural Science Foundation of China Supervision Committee also confirmed misconduct and concluded that Xu Zekuan is responsible for the issues identified. The data provided by the authors was insufficient to resolve the concerns. Accordingly, the Editorial Decision has been made to retract the paper. The authors have been notified about the decision.

Original article: Oncotarget. 2017; 8:51177–51189. 51177-51189. https://doi.org/10.18632/oncotarget.17750
